# Expression of HLA Class II Molecules in Humanized NOD.Rag1KO.IL2RgcKO Mice Is Critical for Development and Function of Human T and B Cells

**DOI:** 10.1371/journal.pone.0019826

**Published:** 2011-05-17

**Authors:** Rebecca Danner, Snehal N. Chaudhari, John Rosenberger, Jacqueline Surls, Thomas L. Richie, Teodor-Doru Brumeanu, Sofia Casares

**Affiliations:** 1 US Military Malaria Vaccine Program, Naval Medical Research Center/Walter Reed Army Institute of Research, Silver Spring, Maryland, United States of America; 2 Department of Medicine, Uniformed Services University of Health Sciences, Bethesda, Maryland, United States of America; Université Paris Descartes, France

## Abstract

**Background:**

Humanized mice able to reconstitute a surrogate human immune system (HIS) can be used for studies on human immunology and may provide a predictive preclinical model for human vaccines prior to clinical trials. However, current humanized mouse models show sub-optimal human T cell reconstitution and limited ability to support immunoglobulin class switching by human B cells. This limitation has been attributed to the lack of expression of Human Leukocyte Antigens (HLA) molecules in mouse lymphoid organs. Recently, humanized mice expressing HLA class I molecules have been generated but showed little improvement in human T cell reconstitution and function of T and B cells.

**Methods:**

We have generated NOD.Rag1KO.IL2RγcKO mice expressing HLA class II (HLA-DR4) molecules under the I-E^d^ promoter that were infused as adults with HLA-DR-matched human hematopoietic stem cells (HSC). Littermates lacking expression of HLA-DR4 molecules were used as control.

**Results:**

HSC-infused HLA-DR4.NOD.Rag1KO.IL-2RγcKO mice developed a very high reconstitution rate (>90%) with long-lived and functional human T and B cells. Unlike previous humanized mouse models reported in the literature and our control mice, the HLA-DR4 expressing mice reconstituted serum levels (natural antibodies) of human IgM, IgG (all four subclasses), IgA, and IgE comparable to humans, and elicited high titers of specific human IgG antibodies upon tetanus toxoid vaccination.

**Conclusions:**

Our study demonstrates the critical role of HLA class II molecules for development of functional human T cells able to support immunoglobulin class switching and efficiently respond to vaccination.

## Introduction

Early models of humanized mice used for HIS reconstitution were generated in the late 1980s by infusion of human peripheral blood mononuclear cells into T- and B-cell deficient rodents such as *scid* mice [1]–[5], a strain derived from CB.17 mice that bears a mutation in the protein kinase, DNA-activated catalytic polypeptide (prkdc) [6]. The level of reconstitution in *scid* mice was however low (<1%) and consisted mainly of human T cells, as very few human B cells were able to survive. These humanized mice also faced the challenge of human T cell functional impairment upon vaccination, and in some cases development of graft versus host disease (GVHD) as a consequence of human T cell reactivity against the mouse MHC antigens [4], [5].

To increase the level of human cell reconstitution and to overcome GVHD, investigators used *scid* mice infused with human hematopoietic stem cells (HSC) [3], [7], [8]. This model known as Hu-HSC-*scid* was able to develop human B and myeloid cells but the life-span of these human cells was relatively short, and the model also failed to develop critical compartments of the human immune system such as T cells. This limitation was mostly attributed to the destructive activity of mouse natural killer (NK) cells, since the development of mouse NK cells is not affected by the *scid* mutation [6]. Inbreeding the *scid* mutation into the non-obese diabetic (NOD) mouse increased the rate of human cell reconstitution upon HSC infusion due to the reduced number and functional impairment of NK cells in the NOD mouse [9], [10]. Yet, the NOD.*scid* mouse model still did not support high rates or long-lasting reconstitution with human immune cells, nor did it allow development of functional human T and B cells [11].

The generation of mice bearing a mutation in the IL2R gamma chain (IL2Rγc) was a major breakthrough in the field, as this strain is devoid of NK cells [12]. Genetic stocks of NOD.*scid*.IL2RγcKO, NOD.RagKO.IL2RγcKO, and BALB/c.RagKO.IL2RγcKO mice were then generated and used as neonatal or adult recipients of human HSCs [13]–[17]. Although both HSC-infused newborn and adult mice were highly reconstituted with human B cells, the development of B cells was arrested in an early stage and did not suffice for reconstitution of human immunoglobulins (natural antibodies) in serum, other than IgM [18]. Newborn recipient mice were much more efficient for development of human T cells than the adult recipients [19], but the human T cells were functionally impaired, as their response to *in vitro* polyclonal T cell stimulation with anti-CD3/CD28 Abs or PMA/ionomycin was poor [18]. Studies from several laboratories further indicated that these mice were sub-optimal, or alternatively failed to elicit cellular and/or humoral responses upon infection or vaccination [15], [20]–[22].

Impairment of human T and B cell function in HSC-reconstituted IL2RγcKO genetic stocks has been attributed to the lack of expression of Human Leukocyte Antigens (HLA) in the mouse thymus, since HLA molecules are required for development of human T cells [23] that in turn, are essential for stimulation of B cells towards immunoglobulin class switching and antibody secretion [24]. Indeed, mice transplanted with human fetal thymus and liver under the kidney capsule and at the same time infused with human HSCs (a model known as BLT mouse –Bone marrow, Liver, Thymus) [25] showed significant improvement in the function of human T and B cells, as these mice were able to elicit specific IgM responses upon vaccination [25], [26].

Recently, humanized mice expressing HLA class I (HLA-A2) molecules on the NOD.*scid*.IL2RγcKO background have been generated [27]–[29]. Contrary to expectations, the HLA-A2 transgenic (Tg) mice showed similar rates of human T cell reconstitution as non-Tg littermates [28], though some of the T cell reconstituted mice were able to elicit HLA-A2-restricted CD8 T cell responses upon infection with Epstein Barr or dengue virus [27]–[29]. However, the function of human B cells was not improved by the transgenic expression of HLA-A2 molecules in HSC-recipient mice, as these mice were unable to elicit specific IgG antibodies [27]–[29].

Herein, we show that a newly-generated strain of NOD.Rag1KO.IL2RγcKO mice expressing HLA-DR*0401 molecules (hereafter referred to as DRAG mice) develop high rate of human T and B cell reconstitution upon infusion of HLA-DR-matched HSC during adulthood. Both CD4^+^ and CD8^+^ human T cell subsets as well as human B cells are highly functional as indicated by their ability to secrete human cytokines, reconstitute serum levels of human IgM, IgG, IgA, and IgE, and elicit specific IgG antibodies upon vaccination. The results demonstrate that expression of HLA class II molecules in humanized mice supports the development of a functional human adaptive immune system.

## Results

### DRAG mice infused with HLA-DR-matched HSC are highly reconstituted with human T cells

Early lymphoid precursors derived from hematopoietic stem cells in the bone marrow migrate to the thymus where they differentiate into CD4^+^CD8^+^ double positive (DP) cells. DP cells undergo positive or negative selection upon recognition of self-peptides presented by thymic epithelial cells in the context of MHC molecules [23], with positively-selected DP thymocytes differentiating into CD4^+^ or CD8^+^ single positive (SP) thymocytes that then repopulate peripheral lymphoid organs.

Thymic selection is a physiological process that prevents autoimmunity by eliminating self-reactive T cells and at the same time generates a broad spectrum of T cells reactive against foreign antigens.

**Figure 1:**
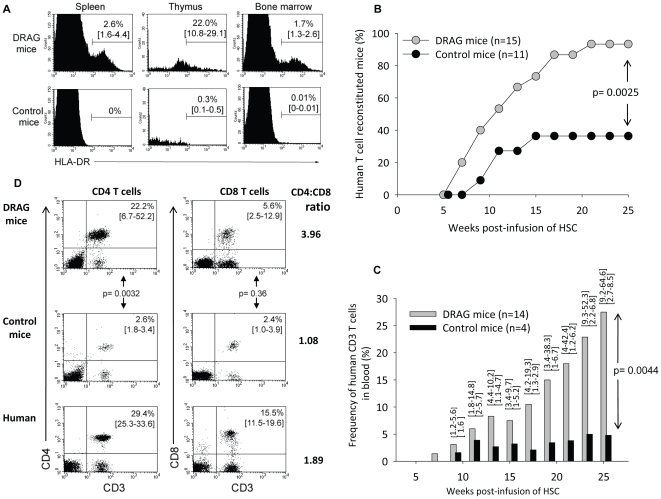
Kinetics of human T cell reconstitution in blood of DRAG mice. Panel A, FACS analysis of splenic, thymic, and bone marrow cells from naïve (non-HSC infused) DRAG and control mice (n = 3) stained with HLA-DR antibodies. Groups of DRAG (n = 15) and control (n = 11) mice that were infused intravenously with 80,000 enriched-HSC (CD34^+^) cells from umbilical cord blood of HLA-DR4 (*0401) newborns were examined in peripheral blood by FACS using anti-human CD3, CD4, and CD8. Panel B, percentage of human T cell reconstituted mice at various time points post-infusion of HSC. Fisher exact test indicated that the rate of human T-cell reconstitution in DRAG mice (14 out of 15) was significantly superior to that in control mice (4 out of 11) (p = 0.0025). Panel C, frequency of human CD3^+^ T cells in blood of reconstituted DRAG (n = 14) and control (n = 4) mice. The cut-off for positive human CD3^+^ T cells was calculated as three times the standard deviation over the background levels of cells from naïve (non-HSC infused) DRAG mice that were stained with anti-human CD3 (>0.17%). Ranges are indicated over the plots. Mann-Whitney test indicated that the frequency of human T cells in blood of DRAG mice was significantly higher than that in control mice (p = 0.0044). Panel D, frequency of human CD4 and CD8 T cells in blood of T cell reconstituted DRAG mice (n = 14) and control mice (n = 4) at 25 weeks post-infusion of HSC (upper two panels). Lower histograms shows frequencies of CD4 and CD8 T cells in human blood (n = 2). P values were calculated using the Mann-Whitney test.

To test the hypothesis that constitutive expression of HLA class II molecules in humanized mice rescues development of human CD4 T cells, we generated NOD.Rag1KO.IL2RγcKO mice expressing HLA-DR4 (0401) molecules (hereafter referred to as DRAG mice) (Fig. 1A upper panel). Four-to-six week-old DRAG mice were infused with CD34^+^ HSC from HLA-DR*0401-positive umbilical cord bloods and monitored by FACS for development of human T cells in the peripheral blood. As a specificity control, we used NOD.Rag1KO.IL2RγcKO littermates lacking expression of HLA-DR4 molecules (Fig. 1A, lower panel) that were infused with HSC from the same donors. As illustrated in figure 1B, the rate of human T cell reconstitution in DRAG mice was considerably higher than in control mice, since 14 out of 15 (93.3%) DRAG mice developed human T cells by 25 weeks post-infusion with HSC as compared to only 4 out of 11 (36.4%) control littermates developing human T cells. In addition, the frequency of human T cells in the blood of reconstituted DRAG mice (n = 14) was considerably higher than in the reconstituted control littermates (n = 4) (Fig. 1C, p = 0.0044, Table S1). Thus, the results demonstrated that expression of HLA-DR4 molecules in DRAG mice promoted development of human T cells.

T cell-reconstituted DRAG (n = 14) and control mice (n = 4) developed both CD4 and CD8 T cells in blood, but the frequency of CD4 T cells was significantly higher in DRAG mice (Fig. 1D, p = 0.0032). Of note, the frequency of blood circulating CD4 T cells in DRAG mice was comparable to that of human volunteers (Fig. 1D, upper and lower panels). On the other hand, the frequency of human CD8 T cells in blood of both groups of mice was similar (Fig. 1D, p = 0.36) and at the same time lower than that in blood of human volunteers.

**Figure 2:**
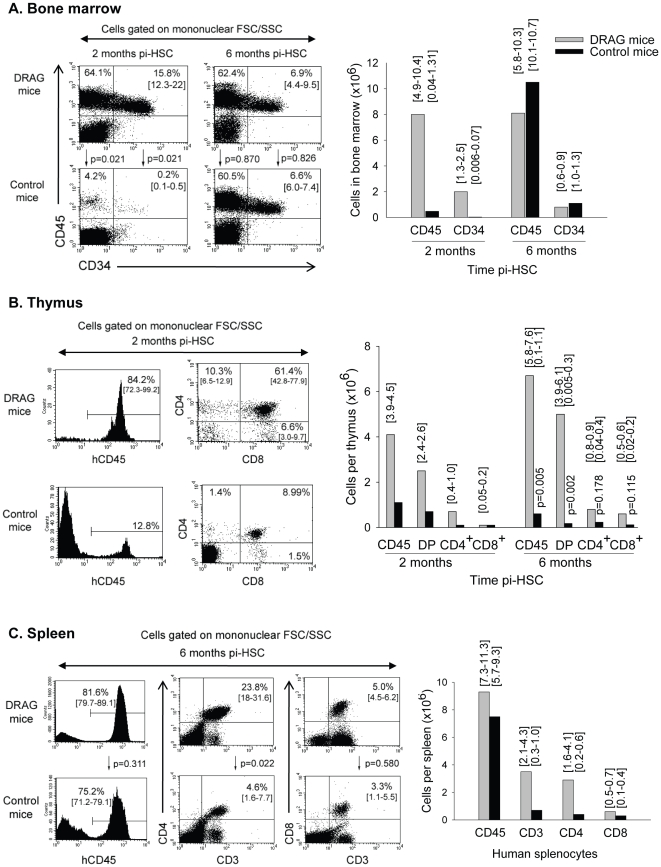
Kinetics of human T cell reconstitution in peripheral lymphoid organs. Groups of DRAG and control mice (n = 4) were euthanized at 2 months post-infusion of HSC and bone marrow (panel A) and thymus (panel B) were examined for the presence of human cells by FACS using specific antibodies. All DRAG mice reconstituted bone marrow and thymus with human cells. All control mice reconstituted bone marrow with human cells, though at much lower frequencies than DRAG mice, and only 1 of them was able to reconstitute thymus with human cells. No significant human T cell reconstitution was detected in spleens of DRAG or control mice at this time point (data not shown). Additional groups of DRAG (n = 4) and control (n = 2) mice that showed human T cells in blood at 6 months post-infusion of HSC, were examined by FACS in bone marrow (panel A), thymus (panel B) and spleen (panel C). Data represent mean values of mice analyzed individually. Ranges are indicated over the plots. P values were calculated using Independent Samples t-test. Of note, control mice (n = 3) that did not have human T cells in blood, also showed absence of human T cells in thymus and spleen (data not shown).

Since T cells are developed in thymus from bone marrow precursors, we next investigated the kinetics of human cell reconstitution in bone marrow, thymus, and spleen of DRAG mice at 2 and 6 months post-infusion of HSC. At 2 months post-infusion of HSC, the bone marrow of DRAG mice (n = 4) showed much higher numbers of CD45^+^ and CD34^+^ cells than the control mice (n = 4) (Fig. 2A). CD45 is a type I transmembrane protein expressed in all human hematopoietic cells except erythrocytes and plasma cells, and CD34 is a marker specific for human HSCs. The thymus of DRAG mice also showed considerably higher numbers of human CD45^+^ cells than the control mice, with most CD4^+^CD8^+^ DP, followed by CD4^+^ SP and CD8^+^ SP (Fig. 2B). Of note, all DRAG mice (n = 4) used for these analyses had human cells in thymus but only 1 out of 4 control mice had human cells in thymus. Thus, these results indicated that expression of HLA-DR4 molecules in DRAG mice favored HSC engraftment in bone marrow and more importantly, promoted the development of human thymocytes.

At 6 months post-infusion of HSC, the bone marrow of DRAG and control mice contained similar numbers of human CD45^+^ and CD34^+^ cells (Fig. 2A), but still the thymuses from control mice were poorly reconstituted with human thymocytes (Fig. 2B, right panel). Furthermore, the spleens of all DRAG mice examined (n = 4) contained mature human T cells (Fig. 2C), of which most were CD4 T cells (23.8%) and, to a lower extent, CD8 T cells (5.0%) (CD4∶CD8 average = 5.2). The frequency of CD4 and CD8 T cells in human spleen is approximately 17% and 14%, respectively [30]. On the other hand, the control mice (n = 2) that were selected based on the presence of human T cells in blood had human T cells in the spleen as well (Fig. 2C), but the CD4 T cell frequency (4.6%) was considerably lower than that in spleens of DRAG mice (CD4∶CD8 average ratio = 1.5). Also, among control mice showing no reconstitution with human T cells in blood (n = 3) all lacked human T cells in spleen (data not shown). These results demonstrated that expression of HLA-DR4 molecules in DRAG mice promoted repopulation of human T cells in the periphery.

**Figure 3:**
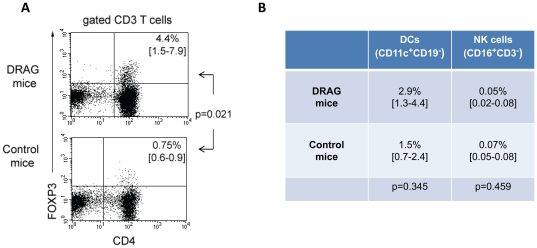
Expression of HLA-DR4 molecules in DRAG mice fosters development of Tregs but not of myeloid and natural killer cells. Panel A, frequency of CD4^+^FOXP3^+^ Tregs among splenic T cells from DRAG (n = 4) and control (n = 2) mice that were examined at 6 months post-infusion of HSC (p = 0.021). Panel B, frequency of dendritic cells (CD11c^+^CD19^−^) and NK cells (CD16^+^CD3^−^) in spleens of DRAG (n = 3) and control (n = 2) mice at 6 months post-infusion of HSC. The cut-off for positive cells was 0.09% for DCs, and 0.01% for NK cells. P values were calculated using Independent Samples t-test.

CD4^+^FOXP3^+^ regulatory T cells (Tregs) are the archetype of immune regulatory cells that are naturally developed in the thymus [31]. Tregs serve as “guardians” of the immune system by maintaining immune homeostasis and at the same time restricting the CD4 and CD8 T-cell responses as well as humoral responses to *self* and non-*self* antigens. The frequency of Tregs in human blood is 2–5% among the CD4 T cell population [32]. At 6 months after infusion of HSC, a much higher frequency (p = 0.021) of human CD4^+^FOXP3^+^ Tregs was detected in spleens of DRAG mice as compared to control mice (Fig. 3A). Thus, our results indicated that a major regulatory cell compartment (human CD4^+^ *FOXP3* ^+^ Tregs) was also reconstituted in DRAG mice upon infusion of HLA-DR-matched HSC.

Dendritic cells (DCs) are critical components of the innate immune system due to their potent ability to process antigen and present immunogenic peptides to T cells for activation [33]. The frequency of DCs in human spleen has been estimated as 2.3% of the mononuclear population [34]. As illustrated in figure 3B, both DRAG and control mice developed human DCs (CD11c^+^CD19^−^). The frequency of human DCs in spleens of DRAG mice (2.9%) was slightly higher than that in control mice (1.5%), though it did not reach statistical significance.

Natural killer (NK) cells are characterized by expression of CD16, an immunoglobulin Fc receptor that allows them to eliminate target cells through a process known as “antibody-dependent-cell-cytotoxicity” or ADCC [35]. The frequency of NK cells in human spleen has been estimated as 15% [30]. As shown in figure 3B, the numbers of human NK cells in both DRAG and control mice were insignificant (0.05% *vs.* 0.07%), which indicated that expression of HLA-DR4 did not improve development of human NK cells.

In aggregate, these results demonstrated that expression of HLA-DR4 molecules in DRAG mice favored engraftment of HSC in bone marrow, homing and development of bone-marrow-derived human T cell precursors in thymus, and proficient repopulation of peripheral lymphoid organs with mature CD4 and CD8 T cells. Expression of HLA-DR4 molecules however did not improve development of human NK cells.

### Human CD4 and CD8 T cells developed by DRAG mice are functional

**Figure 4:**
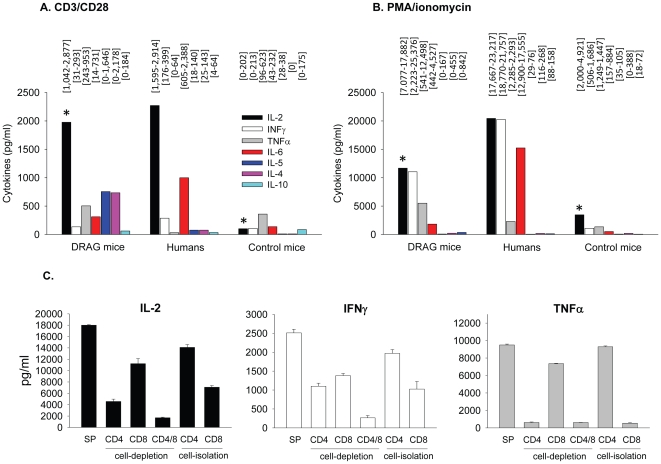
Human T cells developed by DRAG mice are functional. At 6 months post-infusion of HSC, splenic cells (3×10^5^) from DRAG (n = 4) and control mice (n = 2) as in figure 2, were stimulated for 24 h with anti-human CD3/CD28 Abs (panel A) PMA/ionomycin (panel B) or left unstimulated, and cytokines secreted in supernatants were measured by Luminex. Non-stimulated cells secreted insignificant levels of human cytokines (<50 pg/ml). Data represent mean values of mice analyzed individually over the background levels of non-stimulated cultures. Ranges are indicated over the plots. T cells from DRAG mice secreted significantly higher levels of IL-2 upon stimulation with CD3/CD28 Abs or PMA/ionomycin as compared to control mice (p values = 0.013 and 0.025, respectively using the Independent sample t-test). Panel C, cell-depleted spleens or positively isolated CD4 T and CD8 T cells from pooled spleens of two additional DRAG mice were stimulated *in vitro* with PMA/ionomycin for 24 h or left unstimulated. Non-stimulated cell cultures produced insignificant levels of human cytokines (<50 pg/ml). Data represent the mean ± SD of duplicated samples over the background levels of non-stimulated cells. Similar results were obtained in a second independent experiment.

Previous studies in HSC-infused NOD and BALB/c genetic stocks bearing the IL2RγcKO mutation revealed a functional impairment of human T cells reconstituted in these strains, as they responded weakly to polyclonal stimulation with anti-human CD3/CD28 antibodies or PMA/ionomycin [18]. It was thus important to investigate whether expression of HLA-DR4 molecules in DRAG mice may have rescued the function of human T cells. As illustrated in figure 4A&B, human T cells from spleens of DRAG mice (n = 4) responded vigorously to CD3/CD28 or PMA/ionomycin stimulation as indicated by secretion of high levels of human cytokines in cell cultures. Noteworthy, the magnitude of T cell responses in DRAG mice was comparable to that of human PBMCs from healthy volunteers. In contrast, human T cells from the spleens of control mice (n = 2) responded poorly to CD3/CD28 or PMA/ionomycin stimulation (Fig. 4A&B). These results demonstrated that the human T cell compartment reconstituted in DRAG mice is functional.

Since DRAG mice express HLA class II (DR4) molecules but not HLA class I molecules, which is the restriction element for thymic selection of human CD8 T cells, we next compared the function of human CD4 *vs.* CD8 T cells developed by DRAG mice in a well-controlled *in vitro* system. For this comparison, spleen cells from DRAG mice were depleted of either CD4 T cells, CD8 T cells, or both T cell subsets using antibody-coated magnetic beads followed by stimulation with PMA/ionomycin. As illustrated in figure 4C, depletion of human CD4 T cells partially reduced the secretion of IL-2 and IFNγ as compared to that of non-depleted splenic cells, and abrogated secretion of TNFα. Depletion of human CD8 T cells from spleens also reduced the secretion of IL-2 and IFNγ, but had little effect on secretion of TNFα. In addition, positively-isolated CD4 T cells from spleens of DRAG mice secreted high levels of IL-2, IFNγ, and TNFα upon stimulation with PMA/ionomycin, and positively-isolated CD8 T cells secreted IL-2 and IFNγ, but not TNFα. It is know that CD8 T cells secrete little or no TNFα upon PMA/ionomycin stimulation [36]. These results indicated that both human CD4 and CD8 T cells developed by DRAG mice are functional as they respond to stimulation through TCR and non-TCR signaling pathways.

### DRAG mice infused with HLA-DR-matched HSC are highly reconstituted with human B cells

**Figure 5:**
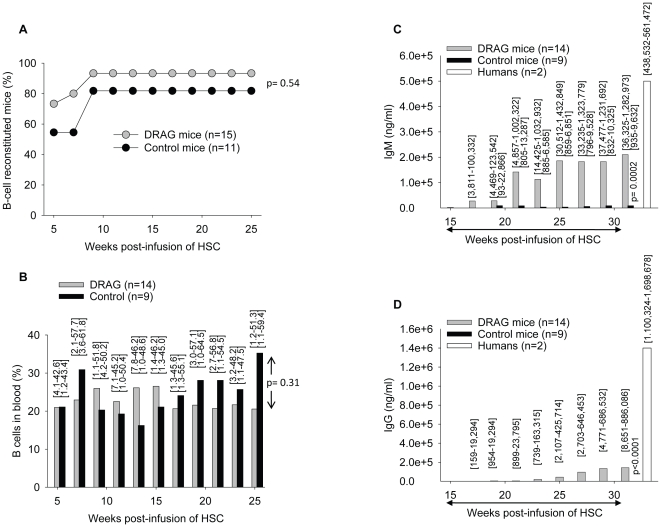
Kinetics of human B cell reconstitution in blood of DRAG and control mice. DRAG (n = 15) and control (n = 11) mice as in figure 1 were examined in peripheral blood by FACS using human CD19 Abs. The cut-off for positive human B cells was calculated as three times the standard deviation over the background of cells from naïve (non-HSC infused) DRAG mice stained with CD19 antibodies (>0.18%). Panel A, percentage of human B cell reconstituted mice at various time points post-infusion of HSC. Fisher exact test indicated that the rate of human B-cell reconstitution in DRAG mice (14 out of 15) was similar to that of control mice (9 out 11) (p = 0.54). Panel B, frequency of human B cells in blood of reconstituted DRAG (n = 14) and control (n = 9) mice (p = 0.31, Mann-Whitney test). Panels C and D, plasma values of human IgM and IgG respectively in B-cell reconstituted DRAG (n = 14) and control (n = 9) mice at various time points post-infusion of HSC (p values 0.0002 and <0.0001, Mann-Whitney test). Data represent mean values of mice analyzed individually. Ranges are indicated over the plots.

The longitudinal FACS analysis in blood of DRAG and control mice using anti-human CD19 revealed that the rate of human B-cell reconstitution was similar between the two strains. Thus, 14 out of 15 (93.3%) DRAG mice and 9 out of 11 (81.8%) control mice were reconstituted with human B (CD19^+^) cells by 10 weeks after HSC infusion (Fig. 5A). Although not statistically significant (p = 0.31), 25 weeks after infusion of HSC the overall frequency of blood circulating human B cells in DRAG mice was slightly lower than in control mice (Fig. 5B, Table S1). Noteworthy, spleens of DRAG mice contained similar numbers of human B cells as compared to control mice (B cell frequency: 47.9%, range [45.9–50] *vs.* 60% [55–65.9]; total number of B cells/spleen: 5.5×10^6^ *±*1.95 *vs.* 6.35×10^6^ *±*2.75, respectively). This indicated that expression of HLA-DR4 molecules in DRAG mice did not improve significantly the reconstitution with human B cells in the peripheral blood on in the spleen as compared to control mice.

### Human B cells developed by DRAG mice are functional and reconstitute plasma levels of all human immunoglobulin classes

**Figure 6:**
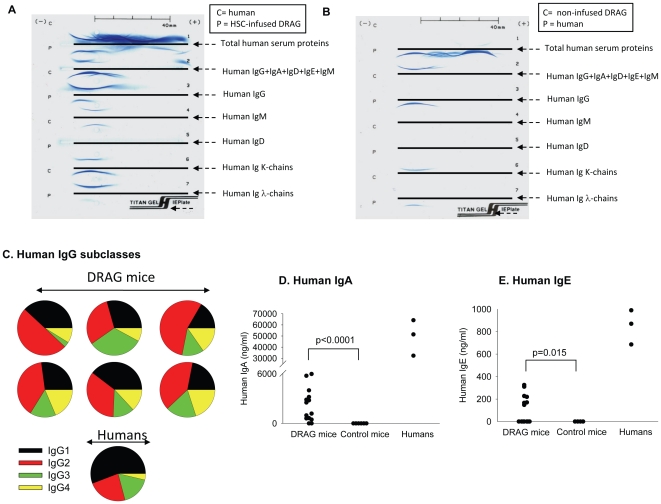
B cells from DRAG mice undergo immunoglobulin class switching. Panels A and B, immunoelectrophoresis of plasma proteins from DRAG mice at 6 months post-infusion of HSC or prior to infusion with HSC, respectively. Panel C, percentage of human IgG subclasses within the total human IgG fraction in plasma of six DRAG mice examined. Panel D, 12 out 14 (85.7%) DRAG mice that showed human B cells in blood, reconstituted human IgA in plasma. Data present mean values of mice analyzed individually. Panel E, 7 out of 14 (50%) DRAG mice also reconstituted human IgE in plasma. No human IgA or IgE was detected in plasma of control mice (p values <0.0001 and 0.015, respectively by Mann-Whitney test).

The major function of B cells is to secrete immunoglobulins (antibodies) that can be detected in blood and body fluids. The function of immunoglobulins is to neutralize and eliminate viruses, bacteria, and parasites as well as to control the homeostatic growth of commensal bacteria in the intestinal tract [24]. In mammalians, there are five classes of immunoglobulins, namely IgD, IgM, IgG, IgA, and IgE, from which IgM and IgG are the most abundant in blood. Previous studies in various humanized mouse models revealed an impaired function of human B cells reflected by poor reconstitution of human IgM serum levels and limited ability to undergo immunoglobulin class switch and to secrete immunoglobulins other than IgM [15], [18], [20]–[22]. Thus, we investigated the kinetics of human immunoglobulin reconstitution in blood of DRAG mice. As illustrated in figure 5C, the levels of human IgM in plasma from B cell reconstituted DRAG mice (n = 14) were significantly higher than those from reconstituted control mice (n = 9) (p = 0.0002). In addition, the DRAG mice were able to reconstitute plasma IgG whilst control mice failed to do so (Fig. 5D, Table S1).The results indicated that the mechanism of immunoglobulin class switch was preserved in DRAG mice. The human identity of Ig heavy and light chains in blood of DRAG mice was confirmed by immunoelectrophoresis (Fig. 6A).

**Figure 7:**
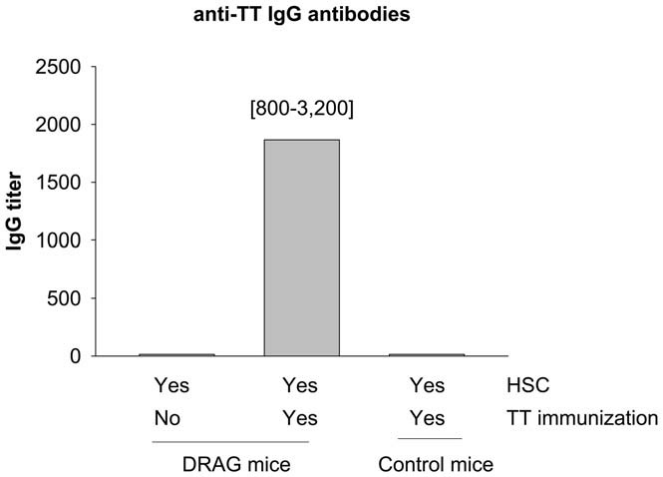
DRAG mice respond to tetanus toxoid (TT) vaccination. Groups of DRAG and control mice (n = 3) were immunized intramuscularly with 1 flocculation unit of TT vaccine, and plasma levels of human anti-TT IgG titers were measured by ELISA at day 14 post-immunization. The frequency of human T and B cells in blood and plasma levels of human immunoglobulins prior to TT-immunization are shown in Table S1.

Human IgG class comprises four different subclasses that differ in structure and biological functions, namely (i) ability to cross placenta, (ii) ability to activate the complement cascade and (iii) affinity binding to Fc receptors expressed on phagocytic cells [37]. As illustrated in figure 6C, DRAG mice reconstituted plasma levels of all human IgG subclasses, with IgG2 as the most prevalent followed by IgG1, IgG3 and IgG4, whereas in the human blood the most prevalent subclass is IgG1, followed by IgG2, IgG3 and IgG4 [36]. In addition, 12 out of 14 (85.7%) DRAG mice reconstituted plasma levels of human IgA (Fig. 6D) and 7 out of 14 (50%) reconstituted plasma levels of human IgE (Fig. 6E). In contrast, control mice did not show detectable levels of human IgA or IgE (Fig. 6D&E, Table S1). The results demonstrated that human B cells developed by DRAG were functional as they secreted all human immunoglobulin classes, whereas those from control mice were impaired in their ability to undergo class switching and secreted only IgM.

### Human B cells develop by DRAG mice respond to tetanus toxoid immunization

A major function of the immune system is to protect against infections by eliciting specific antibodies that bind to and eliminate pathogens. Tetanus toxoid, a formalin-detoxified form of *Clostridium tetani* toxin adjuvanted in alumn, is a licensed human vaccine that induces neutralizing anti-tetanus toxin (TT) IgG antibodies. We investigated whether human B cells from DRAG mice could elicit specific humoral responses upon immunization with TT vaccine. Groups of HSC-infused DRAG and control mice were immunized with 1 flocculation unit of TT vaccine (Sanofi Pasteur) by the intramuscular route, and the titers of anti-TT IgG Abs were measured by ELISA 14 days later. As illustrated in figure 7, immunized-DRAG mice were able to elicit anti-TT IgG antibodies, whereas the control mice failed to do so. These results clearly indicated that human B cells developed by DRAG mice are fully functional. In addition, the results demonstrated that the “human immune system” developed by DRAG mice is superior to that developed by BLT mice, currently referred as the golden standard for humanized mice, since the BLT mice cannot elicit specific IgG antibodies upon TT immunization [26].

## Discussion

The critical role of thymus in T cell development was revealed in the 1930s when a line derived from CB.17 mice, nicknamed “nude” mice due to alopecia, was found athymic and unable to repopulate peripheral lymphoid organs with mature T cells [38]. Subsequent studies demonstrated that surgical removal of the thymus in neonatal mice reduced the numbers of mature T cells in the periphery as well [39]. These studies also demonstrated the critical role of T cells in commanding humoral responses, since both “nude” and thymectomized neonatal mice are impaired in eliciting antibodies, though they develop normal numbers of B cells. Studies by Zinkernagel and others further demonstrated a critical role of MHC molecules in the development and function of T cells through a process of thymic selection [40]. In this study, we demonstrated that transgenic expression of HLA class II (DR4) molecules in NOD.Rag1KO.IL2RγcKO mice rescues the development and function of human T and B cells upon infusion of adult mice with HSC from HLA-DR-matched umbilical cord bloods. The rates of T cell reconstitution and frequencies of human CD4 T cells in DRAG mice were significantly superior to those in control NOD.Rag1KO.IL2RγcKO mice (lacking HLA-DR4 molecules) that were infused with HSC from the same donors. The significant difference between DRAG and control mice was solely associated with expression of HLA-DR4 molecules. The HLA-DR4 molecules thus promoted (i) efficient human HSC engraftment in bone marrow, (ii) thymic development of bone marrow-derived human pro-T cells, (iii) proficient thymic selection of human thymocytes, and (iv) export of high numbers of terminally differentiated human CD4 T cells and Tregs to the periphery.

There is evidence indicating that administration of anti-MHC class II antibodies prevent bone marrow engraftment in dogs [41] and inhibit differentiation of cultured human HSCs *in vitro* [42]. Furthermore, human studies showed that HLA-DR compatibility increases the engraftment efficiency in bone marrow transplants [43]. As shown in our study, expression of HLA-DR4 molecules in the bone marrow of DRAG mice favored early engraftment and expansion of HLA-DR-matched HSC, while control mice lacking expression of HLA-DR4 and infused with the same HSCs failed to do so. The mechanisms by which HLA-DR4 expression in the mouse bone marrow environment promote early engraftment of human HSC remains to be investigated.

Our results also argue against the hypothesis that the adult mouse thymus, which is characterized by a loss of the structural and hormonal milieu, is unable to sustain development and differentiation of human T cells [19]. This is because the adult thymus of HSC-infused DRAG mice allowed engraftment and development of a large number of human thymocytes and exported mature CD4 T cells to periphery. Of note, the low rate of human T cell reconstitution in our control (NOD.Rag1KO.IL2RγcKO) mice (36%) was similar to that reported in studies using NOD.*scid*.IL2RγcKO mice [18], [19], which further validates the superiority of DRAG mice for reconstitution of human T cells.

Almost all DRAG mice (14 out of 15) developed human CD8 T cells as compared to only 4 out 11 control mice. This was an interesting finding, since DRAG mice do not express HLA class I molecules, the restriction element for thymic differentiation of human CD8 T cells. Recently, NOD.*scid*.IL2RγcKO mice expressing HLA class I (A2) molecules have been generated [27]–[29]. Interestingly enough, the transgenic expression of HLA-A2 molecules did not improve the rates of human CD8 T cell reconstitution as compared to non-transgenic littermates, though the human CD8 T cells developed by some of the transgenic mice were restricted by HLA-A2 molecules. Thus, expression of HLA class I molecules does not suffice for promoting engraftment of pro-T cells derived from bone marrow and efficient development of human T cells, whilst expression of HLA class II does. The superiority of DRAG mice in developing human CD8 T cells as compared to our control mice and HLA-A2 Tg mice [27]–[29] suggests the possibility that human CD8 T cells developed by DRAG could have been educated and restricted by HLA class II molecules. This remains to be further investigated.

Secondly, human T cells developed by DRAG mice were as functional as those from healthy human volunteers, since they vigorously responded to CD3/CD28 cross-linking and PMA/ionomycin stimulation by secretion of human Th1 and Th2 cytokines (IL-2, IFNγ, TNFα, IL-6, IL-5, and IL-4), whereas human T cells from control mice failed to do so. Our results in control mice are in agreement with numerous studies on NOD.*scid*.IL2RγcKO mice showing human T cell functional impairment [15], [18], [20]–[22]. CD3/CD28 Abs stimulate T cells by crosslinking TCR and CD28 co-receptor followed by activation of ZAP70 intracellular signaling pathway that ultimately leads to the transcriptional activation of genes involved in T cell differentiation and cytokine secretion [44]. On the other hand, PMA/ionomycin stimulates T cells by inducing calcium influx, thereby bypassing TCR signaling pathways [45]. Our results clearly indicated that expression of HLA-DR4 molecules in DRAG mice promoted the development of human CD4 and CD8 T cells with preserved integrity of both TCR and non-TCR signaling pathways.

It has been suggested that low frequency and functional unresponsiveness of human T cells from humanized IL-2RγcKO genetic stocks lacking expression of HLA class II molecules maybe due to a high level of FAS receptor (CD95) expression on T cells, thereby increasing susceptibility to T cell death [18]. We found that the level of CD95 expression on reconstituted human T cells from DRAG and control mice was similar (MFI CD3 T cells: 20.55±1.06 *vs.* 18.4±0.42; CD4 T cells 22±1.55 vs. 19.1±0.98; CD8 T cells: 19.2±0.98 *vs.* 15.9±1.94, respectively). Therefore, it is unlikely that Fas-FasL induced apoptosis plays an important role in elimination of mature T cells, which might account for the low rates of T cell reconstitution and T cell numbers in our control mice or other similar humanized mouse strains lacking expression of HLA-DR4 molecules. Rather, the low human T cell reconstitution rate in our control mice is attributed to inefficient engraftment of pro-T cells derived from bone marrow and poor thymic selection of human thymocytes.

Thirdly, though expression of HLA-DR4 molecules in DRAG mice did not affect the reconstitution levels with human B cells as compared to control mice, the B cells developed by DRAG mice were functional and able to reconstitute serum levels of human IgM comparable to those in human sera, whereas the levels of human IgM in control mice were significantly lower (p = 0.0002). More importantly, the human B cells from DRAG mice were able to undergo immunoglobulin class switching and to secrete IgG, IgA, and IgE. In contrast, the B cells from our control mice, and other similar genetic stocks [15], [18], [20]–[22] could only secrete low levels of human IgM and were impaired for undergoing immunoglobulin class switching.

Two categories of antigens can activate B cells, and they are defined as T-cell independent or T-cell dependent antigens based on their ability to induce antibodies in athymic (nude) mice that lack mature T cells [24]. T-cell independent antigens are polyclonal B cell activators, such as bacterial polysaccharides and other components of the bacteria cell wall that act mainly through toll-like-receptors (TLR) rather than through the B cell receptor (surface Ig). Humoral responses to T-cell independent antigens are mainly of IgM class as they do not required CD4 T cell help. On the other hand, most antigens are T-cell dependent, meaning that CD4 T cell help is required for immunoglobulin class switching and antibody production. Unlike the T-cell independent antigens, activation of B cells by T-cell dependent antigens requires two signals, the first from interaction of antigen with the B cell receptor and the second from soluble factors secreted by CD4 T cells, such as IL-4, IL-6, or IL-21 [46]–[48]. The role of these cytokines in promoting B cell activation and immunoglobulin class switching has been demonstrated in IL-21R/IL-4 double deficient mice, which can only secrete IgM [49], and also in IL-6 transgenic mice that develop plasmocytosis and hyper-IgG globulinemia [50]. The ability of DRAG mice to generate functional human T cells able to secrete cytokines required for immunoglobulin class switching may account for the development of functional human B cells that reconstituted serum levels of human natural antibodies of all classes and subclasses. Importantly enough, our study also demonstrated that the human immune system developed by DRAG mice elicited specific IgG antibodies upon immunization with tetanus toxoid vaccine. Our vaccinated-control mice were however unable to do so, and this is in agreement with previous studies in IL2RγcKO genetic stocks lacking HLA class II molecules that were also severely impaired for eliciting human IgG responses to vaccination [15], [20]–[22], [26]–[28].

In summary, our results demonstrated that expression of HLA class II molecules in humanized mice is critical for development of functional human T and B cells able to efficiently respond to vaccination. DRAG mice represent a new *in vivo* model for studies on human T and B cell development and function, vaccine testing, and generation of human IgG monoclonal antibodies for prophylactic and/or therapeutic use.

## Materials and Methods

### Ethics Statement

All animal procedures reported herein were conducted under IACUC protocols (ID numbers: D03-07, D06-07 and D07-10) approved by WRAIR/NMRC and in compliance with the Animal Welfare Act and in accordance with the principles set forth in the “Guide for the Care and Use of Laboratory Animals,” Institute of Laboratory Animals Resources, National Research Council, National Academy Press, 1996. The protocol for human use was approved by the Naval Medical Research Center Institutional Review Board in compliance with all applicable Federal regulations governing protection of human subjects. De-identified human blood samples were obtained through commercial vendors.

### DRAG mice

C57BL/6 mice expressing transgenically HLA-DR4 (HLA-DR*0401/I-E^d^) molecules under the mouse I-E^d^ promoter were purchased from Taconic (Hudson, NY) and backcrossed with NOD.Rag1KO mice (The Jackson Labs, Bar Harbor, Maine) for 12 generations. Litters from F2-F12 generation were screened for HLA-DR4/I-E^d^αβ transgenes by PCR using specific primers for HLA-DR4α (forward, AATCCTGACCAATCAGGCGAG, reverse, GCGGAAGAGGTGATCGTCCCT) and HLA-DR4β (forward, GTTTCTTGGAGCAGGTTAAAC; reverse, CTGCACTGTGAAGCTCTCAC) transgenes. Rag1KO litters were identified by the absence of CD3^+^ T cells upon staining of peripheral blood cells with anti-mouse CD3 (Beckton Dickinson, San Jose, CA). The level of inbreeding of F12 mice in NOD background was 100% as determined by microsatellite analysis (Mappairs, Diversified Biopharma Solutions Inc, Loma Linda, CA). F12: HLA-DR4 Tg. NOD.Rag1KO males were crossed with NOD.Rag1KO.IL2RγcKO females (The Jackson Laboratories) and male progeny was screened for HLA-DR4 transgenic (the IL2Rγc gene is located in the X chromosome). Positive males were crossed with NOD.Rag1KO.IL2RγcKO females and all progeny was screened for HLA-DR4αβ transgenes. The HLA-DR4 (+/−) Tg.NOD.Rag1KO.IL2RγcKO (referred as DRAG) mice were maintained in the pathogen-free WRAIR Veterinary Medicine Facility.

### Infusion of mice with HSC

Umbilical cord bloods (UCB) were purchased from AllCells (Emeryville,CA) and Promocell (Heidelberg, Germany). Screening for HLA-DRB1*0401 allele was carried out using DRB1*04 SSP Unitray (Invitrogen, San Diego, CA). HLA-DRB1*0401-positive cord bloods were enriched for CD34^+^ stem cells using EasySep, Human Progenitor Cell Enrichment kit (StemCell Technologies, Vancouver, BC). Prior to enrichment, the frequency of CD34^+^ cells in UCBs was 1.0±0.5% and after enrichment 62.0±8.6%. Mice were irradiated (350 rads), and injected with 8×10^4^ CD34^+^-enriched HSC cells by the intravenous route. For longitudinal analyses, we used groups of DRAG mice (n = 15) and control littermates (n = 11) that were infused with HLA-DR4-positive cord bloods from the same donors. For cross-sectional analyses we used additional groups of mice that were euthanized at 2 months or 6 months post-infusion of HSCs.

### FACS analysis

Blood drawn for the tail vein (20 µl) was collected with heparin-coated capillary tubes (Fisher Scientific, Pittsburgh, PA). Erythrocytes were lysed using ACK lysis buffer (InVitrogen) and white blood cells were incubated with Fcblock (BD Biosciences, San Jose, CA) for 10 minutes at 4°C, and then stained with anti-human CD3, CD4, CD8, and CD19Abs (BD Biosciences) for 30 minutes at 4°C, washed with PBS/1% BSA/0.1% Na azide and analyzed by FACS on the mononuclear FSC/SSC scatter. As control, we used human peripheral blood from adult healthy volunteers that was collected in heparin-coated tubes and treated as above. Mouse splenic and thymic cells were obtained as described [31], [33], [51] and bone marrow cells were obtained by flushing both tibias. Cell populations were quantified by FACS as above using anti-human CD3, CD4, CD8, CD19, CD45, CD34, CD95, CD11c, CD16, HLA-DR (BD Biosciences) and FOXP3 (eBiosiences, San Diego, CA).

### T cell responses

Splenic cells (3×10^5^) from mice or PBMCs (3×10^5^) from volunteers were stimulated for 24 h with anti-human CD3/CD28-coated magnetic beads (InVitrogen), 1 µg/ml PMA plus 50 nM iomycin (Sigma), or left unstimulated. Cytokines in supernatants were measured by Luminex (InVitrogen).

### Cell depletion and isolation

Spleen cells adjusted to 3×10^6^ cells/ml were depleted of CD4 T cells, CD8 T cells, or both cell subsets with antibody-coated magnetic beads (Dynabeads, InVitrogen). The efficiency of cell depletion was >95% as measured by FACS using CD3, CD4, and CD8 Abs. Equal volumes (0.1 ml) of cell depleted cell suspensions or corresponding volumes of positively isolated cells on the magnetic beads were stimulated with PMA/iomycin for 24 h and cytokine secretion in cell culture supernatants were measured by Luminex (InVitrogen).

### ELISA

Peripheral blood from mice or humans was collected in heparin-coated capillaries and plasma samples were obtained by removal of cells by centrifugation. Total plasma immunoglobulins (IgM, IgG, IgA and IgE) were measured by ELISA Quantitation Sets (Bethyl Labs, Montgomery, Texas). Human IgG subclasses were measured using human IgG Subclass Profile ELISA kits (InVitrogen). To determine titers of anti-TT IgG titers, ninety-six-well plates were coated with 10 µg/ml of tetanus toxin (Calbiochem, La Jolla, CA) in carbonate buffer for 2 h at RT. Plates were washed (ELISA Wash Solution, Bethyl Labs), blocked (ELISA Blocking solution, Bethyl Labs) for 30 min at RT, washed again, and incubated with plasma samples at serial half dilutions starting at 1∶50. After 1 h incubation at RT, plates were washed and incubated with anti-human IgG-HRP Abs (Bethyl Labs) for 1 h at RT, washed, and revealed with Enzyme substrate (TMB, Bethyl Labs) in dark for 15 minutes. The enzymatic reaction was stopped (ELISA stop solution, Bethyl Labs) and plates were immediately read at 450 nm. After plotting the OD values against plasma dilution, the titers were calculated as the prior greatest dilution over a two-fold standard deviation over the background.

### Immunoelectrophoresis

Visualization of human immunoglobulin classes and immunoglobulin light (kappa and lambda) chains was carried out using IEF kits (Helena Labs, Beaumont, TX).

### Immunization with tetanus toxoid

Tetanus toxid vaccine was obtained from the Naval Hospital, Bethesda, MD. Mice were injected intramuscularly with 1 floculation unit (fl).

### Statistical analysis

Comparison of cell reconstitution rates was carried out using Fisher's exact test. Comparison of cell frequencies and level of immunoglobulins was determined by Mann-Whitney test. For analysis of low number of samples we used the Independent Samples t-test.

## Supporting Information

Table S1 Human reconstitution in blood of DRAG and control mice upon infusion of HLA-DR*0401 hematopoietic stem cells. (*) human cell frequencies measured at 25 weeks post-infusion of HSC. (^#^) concentration of plasma levels of human IgM and IgG (32 weeks post-infusion of HSC) and IgA and IgE (25 weeks post-infusion of HSC). (^&^) mice were immunized with TT at week 31 post-infusion of HSC. (ND) not done.
(DOCX)
