# Supplementary material for: Expression of HLA Class II Molecules in Humanized NOD.Rag1KO.IL2RgcKO Mice Is Critical for Development and Function of Human T and B Cells
Source: PLoS One. 2011 May 17;6(5):e19826. doi: 10.1371/journal.pone.0019826 (PMC3096643; doi:10.1371/journal.pone.0019826)
Supplement: Table S1 — Human reconstitution in blood of DRAG and control mice upon infusion of HLA-DR*0401 hematopoietic stem cells. (*) human cell frequencies measured at 25 weeks post-infusion of HSC. (#) concentration of plasma levels of human IgM and IgG (32 weeks post-infusion of HSC) and IgA and IgE (25 weeks post-infusion of HSC). (&) mice were immunized with TT at week 31 post-infusion of HSC. (ND) not done. (DOCX) [file pone.0019826.s001.docx]

**Table S1. Human reconstitution in blood of DRAG and control mice upon infusion of HLA-DR*0401 hematopoietic stem cells**

| **Mouse#** | **Donor** | **T cells^*^**  **(%)** | **B cells^*^**  **(%)** | **IgM^#^**  **(ng/ml)** | **IgG^#^**  **(ng/ml)** | **IgA^#^**  **(ng/ml)** | **IgE^#^**  **(ng/ml)** | **TT-IgG^&^**  **(titer)** |
| --- | --- | --- | --- | --- | --- | --- | --- | --- |
| DRAG#1 | A | 64.6 | 5.1 | 1,282,973 | 886,086 | 3,954 | 0 | 1,600 |
| DRAG#2 | A | 10.0 | 11.9 | 401,641 | 456,563 | 3,161 | 325 | 3,200 |
| DRAG#3 | B | 9.2 | 41.0 | 36,325 | 20,412 | 5,911 | 148 | 800 |
| DRAG#4 | B | 30.1 | 7.3 | 49,137 | 11,210 | 600 | 0 | ND |
| DRAG#5 | C | 23.9 | 13.5 | 161,405 | 78,000 | 456 | 0 | ND |
| DRAG#6 | C | 0 | 0 | 0 | 0 | 0 | 0 | ND |
| DRAG#7 | C | 16.3 | 2.1 | 81,258 | 169,080 | 0 | 166 | ND |
| DRAG#8 | C | 34.5 | 35.7 | 95,411 | 59,000 | 959 | 0 | ND |
| DRAG#9 | C | 18.8 | 14.3 | 64,099 | 144,000 | 1,170 | 310 | ND |
| DRAG#10 | C | 49.1 | 1.2 | 133,144 | 85,900 | 0 | 0 | ND |
| DRAG#11 | D | 42.5 | 5.4 | 53,129 | 9,864 | 5,700 | 0 | ND |
| DRAG#12 | D | 16.6 | 41.3 | 36,612 | 50,212 | 629 | 219 | ND |
| DRAG#13 | D | 15.3 | 51.3 | 55,952 | 10,523 | 2,500 | 0 | ND |
| DRAG#14 | D | 27.9 | 34.3 | 462,602 | 9,477 | 2,763 | 227 | ND |
| DRAG#15 | D | 26.5 | 22.4 | 66,184 | 8,651 | 2,832 | 170 | ND |
| Control#1 | B | 0 | 1.1 | 6,585 | 0 | 0 | 0 | ND |
| Control#2 | B | 2.7 | 2.0 | 6,851 | 0 | 0 | 0 | 0 |
| Control#3 | A | 0 | 35.4 | 2,314 | 0 | 0 | 0 | ND |
| Control#4 | A | 0 | 30.0 | 2,520 | 0 | 0 | 0 | ND |
| Control#5 | A | 4.5 | 55.0 | 935 | 0 | 0 | 0 | ND |
| Control#6 | C | 0 | 0 | 0 | 0 | 0 | 0 | ND |
| Control#7 | D | 8.5 | 59.4 | 9,632 | 0 | 0 | 0 | 0 |
| Control#8 | D | 3.9 | 45.1 | 9,582 | 0 | 0 | 0 | ND |
| Control#9 | D | 0 | 49.2 | 6,263 | 0 | 0 | 0 | ND |
| Control#10 | D | 0 | 26.1 | 8,687 | 0 | 0 | 0 | 0 |
| Control#11 | D | 0 | 0 | 0 | 0 | 0 | 0 | ND |

(*) human cell frequencies measured at 25 weeks post-infusion of HSC. (^#^) concentration of plasma levels of human IgM and IgG (32 weeks post-infusion of HSC) and IgA and IgE (25 weeks post-infusion of HSC). (^&^) mice were immunized with TT at week 31 post-infusion of HSC. (ND) not done
